# Vitamin D Receptor Gene Polymorphisms and Autoimmune Thyroiditis: Are They Associated with Disease Occurrence and Its Features?

**DOI:** 10.1155/2019/8197580

**Published:** 2019-08-21

**Authors:** Adam Maciejewski, Michał J. Kowalczyk, Waldemar Herman, Adam Czyżyk, Marta Kowalska, Ryszard Żaba, Katarzyna Łącka

**Affiliations:** ^1^Department of Endocrinology, Metabolism, and Internal Diseases, Poznan University of Medical Sciences, Poznan, Poland; ^2^Department of Dermatology and Venereology, Poznan University of Medical Sciences, Poznan, Poland; ^3^Outpatient's Unit of Endocrine Diseases, Wschowa, Poland; ^4^Department of Gynecological Endocrinology, Poznan University of Medical Sciences, Poznan, Poland; ^5^Laboratory of Neurobiology, Department of Neurology, Poznan University of Medical Sciences, Poznan, Poland

## Abstract

**Purpose:**

Vitamin D, besides its role in calcium-phosphorus metabolism, turned out to play a significant immunomodulating function. Until now four single nucleotide polymorphisms of vitamin D receptor gene (*VDR*), rs2228570 (*Fok*I), rs1544410 (*Bsm*I), rs7975232 (*Apa*I), and rs731236 (*Taq*I), have been studied in autoimmune thyroid disorders, with conflicting results. Another functional polymorphism of the* VDR* gene, rs11568820 (Cdx2), has been shown to influence the immune system, although it has not been studied for its association with autoimmune thyroiditis to date. Therefore, the study aimed to evaluate the association of these five* VDR* gene polymorphisms with susceptibility to autoimmune thyroiditis among Caucasian Polish population. A relationship between the studied polymorphisms and selected clinical features of the disease was additionally assessed.

**Methods:**

223 patients with autoimmune thyroiditis and 130 control subjects were enrolled in the study.* VDR* polymorphisms were studied by PCR-RFLP or TaqMan real-time PCR.

**Results:**

Allele and genotype distributions of any of the studied polymorphisms did not differ significantly between patients and controls. Similarly, frequencies of haplotypes derived from rs1544410-rs7975232-rs731236 (*Bsm*I-*Apa*I-*Taq*I) polymorphisms were not significantly different in the two studied groups. However, a weak association between rs1544410 (*Bsm*I) or rs7975232 (*Apa*I)* VDR* polymorphisms and thyroid volume was found (p = 0.03 and p = 0.04, resp.).

**Conclusions:**

Our results suggest that* VDR* gene is not a major susceptibility factor for autoimmune thyroiditis development, at least in Caucasian Polish population.

## 1. Introduction

Autoimmune thyroid diseases (AITDs) are common pathologies that affect up to 5% of the population and their incidence is still increasing [1]. Autoimmune thyroiditis (AIT), also known as Hashimoto's thyroiditis, is the most frequent manifestation of AITDs. The etiology of the disease is complex. It develops as a result of an interaction between predisposing gene variants and environmental triggers. Among the known genetic factors there are HLA class II gene variants and polymorphisms of non-HLA immune-regulating genes (e.g.,* CTLA-4*,* FOXP3*, and* PTPN22*) or thyroid-specific genes (*TPO*) [2]. According to twin studies, genetic factors in general predominate and contribute 70-80 % to the development of AIT. However, these already proven genetic agents are responsible for less than 20% of the genetic susceptibility to the disease [3].

The active form of vitamin D was found to exert an immunomodulating effect. Because of that 1,25(OH)_2_D may be a meaningful player in the pathogenesis of autoimmune and inflammatory disorders [4, 5]. Vitamin D modifies cytokine secretion pattern, decreases proinflammatory cytokines production, and increases anti-inflammatory signaling [6]. It was also found that vitamin D can alter the expression of MHC class II and costimulatory molecules on the surface of antigen presenting cells (APCs) [6]. T cells can be affected by vitamin D both directly and through its effect on APCs [7]. Vitamin D also inhibits differentiation of B cells, their proliferation, and immunoglobulin production [6]. 1,25(OH)_2_D downregulates Th17 lymphocytes activity and IL-17 production that are factors potentially affecting the development of autoimmunity [8]. Moreover, an association between vitamin D and regulatory T cells was proven [9]. All these actions of vitamin D seem to induce more tolerogenic phenotype of immune cells.

The biological action of 1,25(OH)_2_D is mediated by the nuclear receptor (vitamin D receptor (VDR)), which is a ligand-activated transcription factor. It regulates several target genes, including those affecting the immune system [10]. Moreover,* VDR* is expressed among others in monocytes, dendritic cells, and activated B or T lymphocytes [11]. It is postulated that different polymorphic variants of* VDR*, through their effect on receptor structure or expression, could influence the risk of autoimmune disorders [4]. To date, four single nucleotide polymorphisms (SNPs) of* VDR* gene have been most extensively studied in AIT patients: rs2228570 (*Fok*I) in exon 2, rs1544410 (*Bsm*I) and rs7975232 (*Apa*I) in intron 8, and rs731236 (*Taq*I) in exon 9. Their role has been confirmed in some autoimmune diseases, for example, type 1 diabetes (T1D), rheumatoid arthritis (RA), or multiple sclerosis (MS) [12]. Rs2228570 is known to be a functional SNP. In the case of rs1544410, rs7975232, and rs731236 functional status is questionable, although these three SNPs reliably represent the largest haplotype block of* VDR* gene [13]. The results obtained so far in AIT are heterogeneous and there is no clear conclusion about the role of these* VDR* variants in the development of thyroid autoimmunity [14–16]. Another candidate SNP of* VDR* that has not been yet studied in AIT is rs11568820 (Cdx2). This functional SNP, located in the promoter region of* VDR*, has been found to affect the immune system [17]. Therefore, the study aimed to assess the relationship between these five* VDR* SNPs (rs2228570, rs1544410, rs7975232, rs731236, and rs11568820) and AIT susceptibility in the Caucasian Polish population. The association between these SNPs and selected clinical features of the disease was additionally analyzed.

## 2. Subjects and Methods

### 2.1. Subjects

223 unrelated adult AIT patients of Caucasian Polish origin were enrolled in the study. They were recruited from endocrinology outpatient clinics over the period of two years, from January 2016 to September 2017. The diagnosis of AIT was made on the basis of standard criteria that include at least three from (1) clinical symptoms of hypothyroidism, (2) hypo- or euthyroidism found by thyroid function tests (TSH), (3) elevated anti-thyroid antibodies level (thyroid peroxidase antibodies (TPOAb) and/or thyroglobulin antibodies (TgAb)), and (4) thyroid ultrasound findings typical for AIT. In a subgroup of patients the diagnosis was confirmed by fine needle aspiration biopsy (FNAB). All patients were diagnosed with hypothyroidism (overt or subclinical dysfunction). However, on enrollment, they were euthyroid (TSH values were within the normal range) on levothyroxine supplementation for at least three months.

Control group consisted of 130 unrelated healthy volunteers of Caucasian Polish origin, predominantly blood donors. They were recruited within the same geographical location as patients. In the control group thyroid gland disorders were excluded by anamnesis, thyroid ultrasonography, and laboratory tests (TSH, TPOAb, and TgAb). None of them had any known family history of AITDs. Basic characteristics of patients and controls are presented in Table 1. History of any other autoimmune, neoplastic, or chronic inflammatory disorder led to exclusion from the study in both patient and control groups. All participants gave written informed consents. The study protocol was approved by the ethics committee of the Poznan University of Medical Sciences (approval number: 443/15).

Detailed thyroid ultrasound (US) reports were available in a subgroup of 98 AIT patients. These patients were further subdivided into AIT with nodules (at least one solid lesion ≥ 5 mm in diameter) and AIT without nodules (AIT patients without any solid lesions ≥ 5 mm in diameter). None of the observed thyroid nodules were autonomous. Patients with nodules suspected for malignancy were referred for FNAB, and those with lesions of Bethesda category II or higher were excluded from further analyses. None of the female patients in this group were pregnant or have delivered in the last 12 months.

### 2.2. Methods

In both patients and controls genomic DNA was isolated from whole blood using spin column-based DNA extraction kits, according to the manufacturer's instructions (NucleoSpin® Blood L kit, Macherey-Nagel).

Rs2228570, rs1544410, rs7975232, and rs731236* VDR* SNPs were genotyped by polymerase chain reaction-restriction fragment length polymorphism (PCR-RFLP). The target DNA fragments were amplified by thermal cycling (MJ Mini Thermal Cycler, Bio-Rad Laboratories) with the use of Color OptiTaq PCR Master Mix (EURx, Poland). PCR products were then digested with appropriate restriction endonucleases. Primer sequences, conditions of the PCR reactions, and the size of products yielded are shown in Table 2 [18–20]. The products of digestion were separated in a 2% agarose gel stained with SYBR® Safe (Thermo Fisher). SNP variants were indicated by (1) nucleotide present in the polymorphic site or (2) the first letter of the restriction enzyme, with uppercase letters in case of the absence and lowercase letters in case of the presence of the cut site.

**Figure 1:**
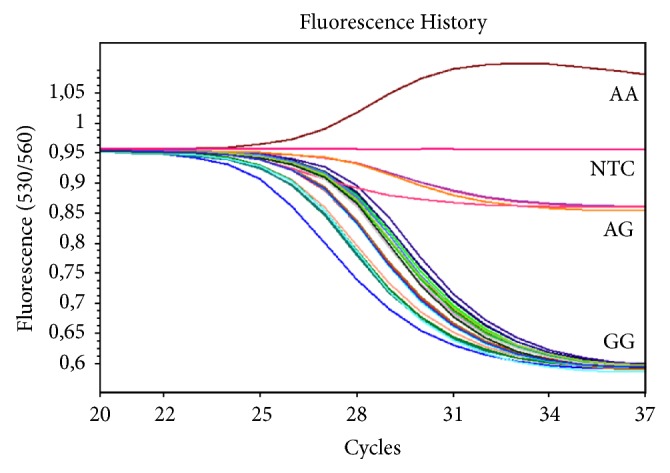
Genotyping the rs11568820 (Cdx2)* VDR* SNP by TaqMan real-time PCR. X axis represents the number of cycles of PCR reaction and the Y axis presents the ratio between the two fluorescence signals. NTC, negative control; AA, AG, and GG, genotypes of rs11568820 SNP.

Rs11568820 genotyping was performed by TaqMan real-time PCR with the use of Roche LightCycler 2.0 thermal cycler. The reaction comprised of Probe qPCR Master Mix E0420 (EURx) and a TaqMan® SNP Genotyping Assay ID: C___2880808_10 (Thermo Fisher). The reaction program was as follows: 37°C, 2 min, Uracil-N-glycosylase pretreatment; 95°C, 10 min, predenaturation; 40x: 92°C, 15 s, ramp rate (RR) 5°C/s; 60°C, 1 min, RR 5°C/s with signal acquisition.  Figure 1 presents a result sample of rs11568820 genotyping.

To confirm the accuracy of the aforementioned methods randomly selected samples were verified by direct sequencing.

In a subgroup of patients thyroid ultrasonography was performed on enrollment, using a ProSound SSD-3500SX ultrasound system with a 7.5 MHz linear transducer (Hitachi Aloka Medical, Japan). The volume of the thyroid gland [ml] was calculated as the sum of the two lobes (each lobe volume estimated with the formula *π*/6 x height [cm] x width [cm] x depth [cm]).

### 2.3. Statistics

The distribution of genotypes in patients and controls was tested for deviations from Hardy-Weinberg equilibrium (HWE) by chi-squared test (*χ*^2^) [21]. The comparison of allele frequencies and genotype distributions between groups was performed by *χ*^2^, Fisher's exact, Fisher-Freeman-Halton, or *χ*^2^ for trend tests, where appropriate. Odds ratio (OR) with 95% confidence interval (95% CI) was calculated as a measure of the strength of association. To compare the clinical parameters between groups and/or subgroups (e.g., age, duration of the disease, and thyroid volume) we used Student's t-test or ANOVA for normally distributed variables and Mann–Whitney U test or Kruskal-Wallis test for variables that were not normally distributed. In the case of significant differences between-groups found by multiple comparison tests (ANOVA or Kruskal-Wallis), post-hoc tests were used for pairwise comparisons. Analyses were performed using GraphPad Prism version 7.03 for Windows (GraphPad Software). A p-value of < 0.05 was considered statistically significant.

Linkage disequilibrium (LD) between studied SNPs was calculated to construct haplotype blocks. In the next step, haplotype frequencies were compared between the groups (with the use of Haploview 4.2 version, Broad Institute) [22].

The numerical data were expressed as mean ± standard deviation or median and interquartile range (for data not normally distributed).

## 3. Results

### 3.1. Allele and Genotype Frequencies

Genotype distributions were consistent with HWE for the studied* VDR* polymorphisms in both groups (p > 0.05). When comparing genotype distributions and allele frequencies of five studied* VDR* SNPs between the AIT group and controls, no statistically significant differences were observed. Allele and genotype frequencies of the studied SNPs and results of the case-control analysis are presented in Table 3.

### 3.2. Haplotype Analysis

According to D' values the strongest LD was observed between three SNPs located in the 3' end of the* VDR* gene: rs731236, rs7975232, and rs1544410. Instead, the weakest LD was found between rs2228570 SNP and studied 3' end polymorphisms (detailed results in Table 4). Haplotype block containing three linked SNPs (rs731236, rs7975232, and rs1544410) was constructed, according to Gabriel et al. [20]. Haplotype analysis was possible in 86.10% of patients and 93.85% of controls. In both the patient and the control groups the most frequent haplotype was TCG (Tab) (49.6% and 50.3%, resp.). The second most frequent haplotype was CAA (tAB), again with similar percentages in both groups (37.1% in AIT and 40.1% in controls). Other haplotypes were rare. Haplotype frequencies did not differ significantly between patients and controls (Table 5).

### 3.3. Association of VDR SNPs with Selected Clinical Features of AIT

The association between* VDR* SNPs and the ultrasonographic features was analyzed in a subgroup of 98 AIT patients. Patients were divided into two subgroups: AIT with nodules and AIT without nodules (as described in Methods Section). These subgroups did not differ significantly in age or gender distribution and time since diagnosis of the disease (p > 0.05). Thyroid volume (TV) was slightly higher in AIT with nodules, as expected (12.65 ± 9.29 vs. 9.38 ± 7.05, p = 0.06). AIT patients with nodules also had lower daily doses of levothyroxine comparing to AIT without nodules group (75 *μ*g (50-100) vs. 100 *μ*g (75-125), p < 0.01). However, we did not observe any significant differences in genotype distributions or allele frequencies of the studied* VDR* SNPs between these two AIT subgroups (data shown in Supplementary Table 1S).

**Figure 2:**
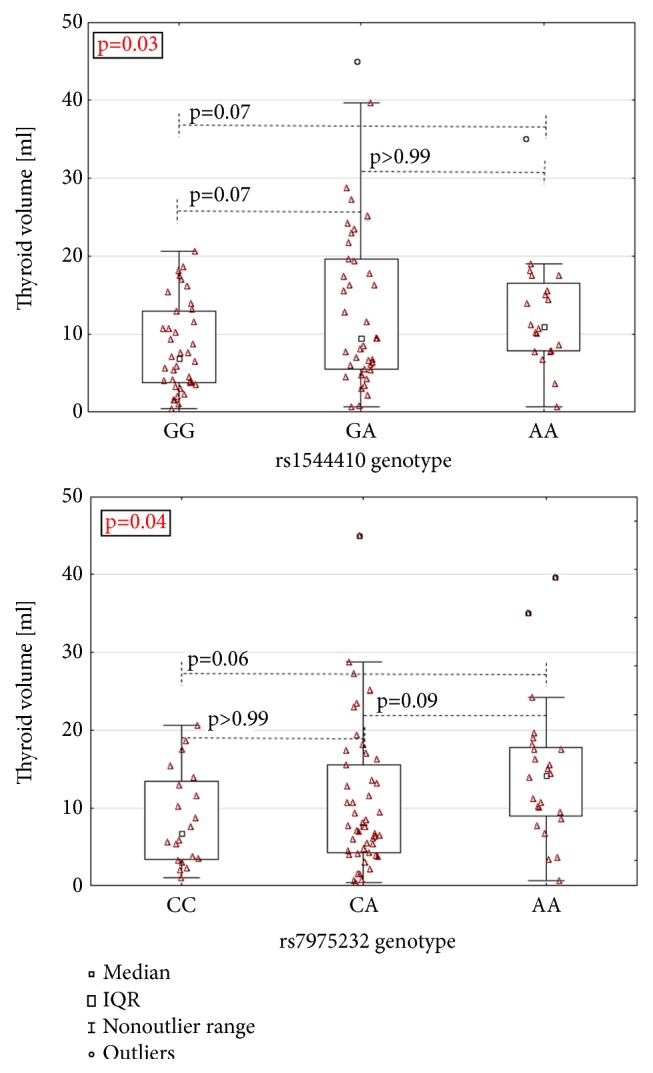
Association between rs1544410 (*Bsm*I) and rs7975232 (*Apa*I) genotypes and thyroid volume in AIT. Median thyroid volume differed significantly according to rs1544410 or rs7975232 genotypes (p-value of Kruskal-Wallis test was 0.03 and 0.04, resp.). Dunn's post-hoc test was performed to show differences between genotype pairs.

The relationship between* VDR* variants and TV in AIT patients was analyzed. It revealed that the TV differed significantly between different genotypes of rs1544410 and rs7975232 (p = 0.03 and 0.04, resp., Figure 2), but not between rs2228570, rs731236, and rs11568820 genotypes (p = 0.29, 0.12, and 0.54, resp.). In the case of rs1544410 and rs7975232, Dunn's post-hoc test was performed for pairwise comparison. It showed that differences between GG and AA and between GG and GA genotypes of rs1544410 were of borderline significance (p = 0.07 in both cases). In the case of rs7975232 SNP the difference between CC and AA genotypes showed a trend toward significance (p = 0.06). The subgroups created according to rs1544410 and rs7975232 genotypes did not differ significantly in age, gender, time since diagnosis of the disease, or daily dose of levothyroxine (p > 0.05 in all cases; for details, see Supplementary Tables 2S and 3S).

We also tested the association between studied* VDR* SNPs and age at AIT diagnosis, although no significant differences were found (data not shown).

## 4. Discussion

*VDR* gene polymorphisms have been extensively studied for their potential association with susceptibility to different autoimmune and inflammatory disorders, for example, MS, T1D, RA, systemic lupus erythematosus (SLE), inflammatory bowel diseases, or tuberculosis [12]. Meta-analyses of different studies confirmed some of these associations, such as rs2228570 and rs731236 in RA [23], rs1544410 and rs731236 in T1D [24], rs7975232 in Crohn's disease, or rs731236 in ulcerative colitis [25]. The interstudy heterogeneity is often reported what may be in part explained by ethnic differences observed in allele and genotype distributions of* VDR* SNPs [26].

In our case-control study of Polish AIT patients, we failed to find any significant associations between rs2228570, rs1544410, rs7975232, and rs731236* VDR* SNPs and the risk of disease development. These results are in accordance with recent European population study from Italy, where no significant difference in* VDR* SNPs distribution between AIT and controls was also found (rs1544410, rs7975232, and rs731236 were assessed) [27]. On the contrary, there are publications where* VDR* SNPs were shown to be associated with AIT in Caucasians; rs2228570 was found to influence the disease risk in Serbian patients and rs1544410 in Croatians [28, 29]. Moreover, two studies from Turkey confirmed the association with rs731236 SNP [30, 31]. In Asians rs2228570 SNP was reported as a risk factor of AIT development in several studies [32–34], although Meng et al. failed to find such an association among the Chinese [35]. In a recent study of Iranian patients, Zarrin et al. showed that rs2228570* VDR* SNP might be weakly associated with the disease risk [36].

In 2013 Feng et al. performed a meta-analysis of available studies on the role of* VDR* SNPs in AITD pathogenesis. They found a relationship between rs1544410 and rs731236 SNP and AITD risk. In Europeans, similarly to overall results, B allele of rs1544410 (*Bsm*I) and t allele of rs731236 (*Taq*I) were associated with reduced AITD risk. Unfortunately, the authors did not perform stratification by AITD subtypes [14]. In 2017 Wang et al. assessed studies on* VDR* SNPs in Hashimoto's thyroiditis and found rs2228570 SNP as a disease risk marker. However, the analysis of subgroups by ethnicity showed no significant differences between patients and controls among Caucasians [15]. In 2018 Gao et al. analyzed the influence of* VDR* SNPs on AITD risk again, adding newly published studies. They found an association between rs2228570 or rs731236 SNPs and AITD. This observation was also confirmed for AIT patients in stratification by AITD subtypes. In Caucasian AITD patients the only significant association was found for rs1544410 SNP [16]. It cannot be excluded that the role of* VDR* gene variants is not the same in two phenotypes of AITD-AIT and Graves' disease. The impact of* VDR* SNPs on predisposition to autoimmunity may also vary depending on studied population or ethnicity.

Rs11568820 (Cdx2)* VDR* SNP was not assessed in AITD patients to date (according to the PubMed database, 31.04.2019). This SNP was previously shown to influence the immune system and modify the risk of some infectious diseases (e.g., tuberculosis and rubella) [17, 37, 38]. However, we did not observe a significant association between rs11568820 SNP and AIT risk. The role of rs11568820 SNP was also studied in different autoimmune diseases, among others in T1D, MS, vitiligo, and psoriasis. Similar to our results, no clear connection with autoimmunity was confirmed [39–42].

In our group of unrelated Polish Caucasians, strong LD was present between three* VDR* SNPs: rs1544410, rs7975232, and rs731236. Rs2228570 and rs11568820 SNPs were not in LD with any of the other studied SNPs. Most frequent haplotypes were TCG (Tab) and CAA (tAB) in both patients and controls, what is in agreement with earlier studies of Caucasian population [27, 43]. We did not confirm the association between* VDR* haplotypes and AIT. Giovinazzo et al. and Meng et al. also did not observe such associations, contrary to the study by Stefanic et al. where the Tab variant was found to confer susceptibility to AIT [27, 29, 35].

Our study showed that there might be a weak association between rs1544410 and rs7975232 SNPs and volume of thyroid gland in AIT patients. The influence of* VDR* SNPs on TV has not been already reported. Meng et al. and Inoue et al. studied the relationship between* VDR* SNPs and goiter degree (evaluated clinically), but they found no associations [32, 35]. Vitamin D level was previously reported to be positively correlated with TV in AIT [44]. One of the possible explanations of the observed associations is that some* VDR* genotypes contribute to the atrophic variant of AIT, in contrast to goitrous form [45]. It was also proven that vitamin D may influence apoptosis of thyrocytes that could lead to volume changes [46]. As TV may vary according to different genetic and exogenous factors [47], aforementioned results must be interpreted cautiously and need further confirmation.

## 5. Conclusions

We found no differences in genotype or allele distributions between AIT patients and the control group for any of the studied* VDR* SNPs. It suggests that* VDR* gene is probably not a strong susceptibility factor for AIT development, at least in the Polish population. It cannot be excluded that association may be minor in case of Caucasian patients comparing to Asians and larger sample size is needed to confirm* VDR* role in determining disease risk.

## Figures and Tables

**Table 1:** Patients and controls characteristic.

	AIT	Healthy controls
n	223	130
Age (SD) [years]	46.88 (13.50)	35.42 (14.96)
Female to male ratio	21.3:1	3.48:1
Thyroid volume (SD) [ml]	11.39 (8.80)	10.22 (4.09)
TSH (SD) [mIU/l]	2.01 (2.08)	1.52 (0.49)
Levothyroxine dose (SD) [*μ*g/kg body mass]	1.19 (0.50)	-
Age at diagnosis (SD) [years]	43.37 (14.57)	-
Follow-up time (IQR) [years]	3 (1-6)	-
Elevated TPOAb [%]	92.83	none
Elevated TgAb [%]	64.13	none

AIT: autoimmune thyroiditis.

Data are mean (SD, standard deviation), median (IQR, interquartile range), or percentage.

**Table 2:** PCR conditions for genotyping of rs2228570 (*Fok*I), rs1544410 (*Bsm*I), rs7975232 (*Apa*I), and rs731236 (*Taq*I) *VDR* SNPs.

SNP	Forward primer	Reverse primer	PCR conditions	Cycles	PCR product size (bp)	Enzyme	Digested products size (bp)
rs2228570	5'-AGCTGGCCCTGGCACTGACTCTGGCT-3'	5'-ATGGAAACACCTTGCTTCTTCTCCCTC-3'	95°C for 5 min; (95°C for 30 s; 63°C for 30 s; 72°C for 30 s); 72°C for 7 min	33	267	*Fok*I	CC: 267 CT: 267, 197, and 70 TT: 197 and 70

rs1544410	5'-ACTTGCATGAGGAGGAGCATGTC-3'	5'-GGAGAGGAGCCTGTGTCCCATTTG-3'	95°C for 5 min; (95°C for 45 s; 61°C for 30 s; 72°C for 45 s); 72°C for 7 min	36	812	*Bsm*I	GG: 475 and 337 GA: 812, 475, and 337 AA: 812

rs7975232 / rs731236	5'-GGTGGGATTGAGCAGTG-3'	5'-ATGCTGCACTCAGGCTG-3'	95°C for 5 min; (95°C for 30 s; 56°C for 30 s; 72°C for 45 s); 72°C for 7 min	35	280	*Apa*I / *Taq*I	*Apa*I: CC: 256 and 24 CA: 280, 256, and 24 AA: 280 *Taq*I: TT: 280 TC: 180 and 100 CC: 280, 180, and 100

SNP, single nucleotide polymorphism; bp, base pair.

**Table 3:** Allele and genotype frequencies of *VDR* SNPs in AIT patients and the control group.

	*AIT* n=223 (%)	*Healthy controls* n=130 (%)	*p*	*OR (95% CI)*
**rs2228570 (*Fok*I)**				

Genotype				

CC (FF)	72 (32.29)	38 (29.23)	0.71 (0.84*∗*)	
CT (Ff)	98 (43.95)	63 (48.46)		
TT (ff)	53 (23.77)	29 (22.31)		

Allele				

C (F)	242 (54.26)	139 (53.46)	0.84	1.03 (0.76-1.40)
T (f)	204 (45.74)	121 (46.54)		

**rs1544410 (*Bsm*I)**				

Genotype				

GG (bb)	86 (39.09)	43 (35.83)	0.51 (0.31*∗*)	
GA (bB)	99 (45.00)	52 (43.33)		
AA (BB)	35 (15.91)	25 (20.83)		

Allele				

G (b)	271 (61.59)	138 (57.50)	0.30	1.19 (0.86-1.63)
A (B)	169 (38.41)	102 (42.50)		

**rs7975232 (*Apa*I)**				

Genotype				

CC (aa)	53 (24.65)	28 (22.40)	0.78 (0.94*∗*)	
CA (aA)	112 (52.09)	70 (56.00)		
AA (AA)	50 (23.26)	27 (21.60)		

Allele				

C (a)	218 (50.70)	126 (50.40)	0.94	1.01 (0.74-1.39)
A (A)	212 (49.30)	124 (49.60)		

**rs731236 (*Taq*I)**				

Genotype				

TT (TT)	79 (36.74)	42 (33.60)	0.63 (0.38*∗*)	
TC (Tt)	106 (49.30)	61 (48.80)		
CC (tt)	30 (13.95)	22 (17.60)		

Allele				

T (T)	264 (61.40)	145 (58.00)	0.38	1.15 (0.84-1.58)
C (t)	166 (38.60)	105 (42.00)		

**rs11568820 (Cdx2)**				

Genotype				

GG	118 (71.08)	91 (76.47)	0.54 (0.39*∗*)	
GA	46 (27.71)	26 (21.85)		
AA	2 (1.20)	2 (1.68)		

Allele				

G	282 (84.94)	208 (87.39)	0.41	1.23 (0.76-2.01)
A	50 (15.06)	30 (12.61)		

AIT, autoimmune thyroiditis; OR, odds ratio; CI, confidence interval; *∗χ*^2^ test for trend.

**Table 4:** Linkage disequilibrium between *VDR* gene polymorphisms.

Locus 1	Locus 2	D' (CI)	LOD	r^2^
**rs731236 (*Taq*I)**	**rs7975232 (*Apa*I)**	1.0 (0.94-1.0)	32.85	0.73
**rs731236 (*Taq*I)**	**rs1544410 (*Bsm*I)**	0.95 (0.89-0.98)	42.13	0.90
**rs731236 (*Taq*I)**	**rs2228570 (*Fok*I)**	0.05 (0.0-0.26)	0.05	0.00
**rs731236 (*Taq*I)**	**rs11568820 (Cdx2)**	0.50 (0.18-0.73)	1.51	0.05
**rs7975232 (*Apa*I)**	**rs1544410 (*Bsm*I)**	0.98 (0.9-1.0)	30.17	0.70
**rs7975232 (*Apa*I)**	**rs2228570 (*Fok*I)**	0.06 (0.0-0.24)	0.08	0.00
**rs7975232 (*Apa*I)**	**rs11568820 (Cdx2)**	0.59 (0.21-0.8)	1.59	0.05
**rs1544410 (*Bsm*I)**	**rs2228570 (*Fok*I)**	0.06 (0.0-0.26)	0.06	0.00
**rs1544410 (*Bsm*I)**	**rs11568820 (Cdx2)**	0.44 (0.14-0.68)	1.29	0.04
**rs2228570 (*Fok*I)**	**rs11568820 (Cdx2)**	0.12 (0.01-0.52)	0.05	0.00

D', value of D prime between the loci; LOD, likelihood odds ratio;

r^2^, correlation coefficient between two loci; CI, confidence interval.

**Table 5:** Haplotype analysis in AIT patients and the control group.

Haplotype	AIT (%)	Healthy controls (%)	p	OR	95% CI
TCG (Tab)	49.6	50.3	0.87	0.97	0.71-1.34
CAA (tAB)	37.1	40.1	0.46	0.88	0.64-1.23
TAG (TAb)	9.7	7.0	0.24	1.43	0.79-2.59
CAG (tAb)	1.6	1.3	0.74	1.25	0.32-4.97

AIT, autoimmune thyroiditis.

The table shows haplotypes with frequencies over 1%.

## Data Availability

The data used to support the findings of this study are included within the article.

## Supplementary Materials

Supplementary Materials Table 1S: Allele and genotype frequencies of* VDR* SNPs in “AIT with nodules” vs. “AIT without nodules.” Table 2S: Characteristic of AIT subgroups according to genotypes of rs1544410 (*Bsm*I)* VDR* polymorphism. Table 3S: Characteristic of AIT subgroups according to genotypes of rs7975232 (*Apa*I)* VDR* polymorphism.
