# Supplementary material for: Vitamin D Receptor Gene Polymorphisms and Autoimmune Thyroiditis: Are They Associated with Disease Occurrence and Its Features?
Source: Biomed Res Int. 2019 Aug 21;2019:8197580. doi: 10.1155/2019/8197580 (PMC6719278; doi:10.1155/2019/8197580)
Supplement: Supplementary Materials — Table 1S: Allele and genotype frequencies of VDR SNPs in “AIT with nodules” vs. “AIT without nodules.” Table 2S: Characteristic of AIT subgroups according to genotypes of rs1544410 (BsmI) VDR polymorphism. Table 3S: Characteristic of AIT subgroups according to genotypes of rs7975232 (ApaI) VDR polymorphism. [file 8197580.f1.zip › Supplement_BMRI_2838758.docx]

Vitamin D receptor gene polymorphisms and autoimmune thyroiditis – are they associated with disease occurrence and its features?

Adam Maciejewski^1&,^ Michał J. Kowalczyk^2&^, Waldemar Herman^3^, Adam Czyżyk^4^, Marta Kowalska^5^, Ryszard Żaba^2^, Katarzyna Łącka^1^

1. Department of Endocrinology, Metabolism and Internal Diseases, Poznan University of Medical Sciences, Poznan, Poland

2. Department of Dermatology and Venereology, Poznan University of Medical Sciences, Poznan, Poland

3. Outpatient’s Unit of Endocrine Diseases, Wschowa, Poland

4. Department of Gynecological Endocrinology, Poznan University of Medical Sciences, Poznan, Poland

5. Laboratory of Neurobiology, Department of Neurology, Poznan University of Medical Sciences, Poznan, Poland

^&^ These authors contributed equally to this work.

Correspondence should be addressed to:

Prof. K. Lacka, MD, PhD

Department of Endocrinology, Metabolism and Internal Diseases

University of Medical Sciences, Poznan, Poland

Tel.: + 48 61 869 1330 Fax: + 48 61 869 1682

[kktlacka@gmail.com](mailto:kktlacka@gmail.com)

**Table 1S.** Allele and genotype frequencies of *VDR* SNPs in “AIT with nodules” vs. “AIT without nodules”.

|  | **AIT with nodules**  n=55 (%) | **AIT without nodules**  n=43 (%) | **p** | **OR (95% CI)** |
| --- | --- | --- | --- | --- |
| **rs2228570 (*Fok*I)** |  |  |  |  |
| Genotype |  |  |  |  |
| CC (FF) | 15 (27.27) | 12 (27.91) | 0.96 (0.93*) |  |
| CT (Ff) | 27 (49.09) | 20 (46.51) |  |  |
| TT (ff) | 13 (23.64) | 11 (25.58) |  |  |
| Allele |  |  |  |  |
| C (F) | 57 (51.81) | 44 (51.16) | 0.93 | 1.03 (0.57-1.84) |
| T (f) | 53 (48.18) | 42 (48.84) |  |  |
| **rs1544410 (*Bsm*I)** |  |  |  |  |
| Genotype |  |  |  |  |
| GG (bb) | 20 (37.04) | 18 (41.86) | 0.62 (1.00*) |  |
| GA (bB) | 24 (44.44) | 15 (34.88) |  |  |
| AA (BB) | 10 (18.52) | 10 (23.26) |  |  |
| Allele |  |  |  |  |
| G (b) | 64 (59.26) | 51 (59.30) | 1.00 | 1.00 (0.57-1.75) |
| A (B) | 44 (40.74) | 35 (40.70) |  |  |
| **rs7975232 (*Apa*I)** |  |  |  |  |
| Genotype |  |  |  |  |
| CC (aa) | 9 (19.15) | 9 (23.68) | 0.64 (0.37*) |  |
| CA (aA) | 24 (51.06) | 21 (55.26) |  |  |
| AA (AA) | 14 (29.79) | 8 (21.05) |  |  |
| Allele |  |  |  |  |
| C (a) | 42 (44.68) | 39 (51.32) | 0.39 | 0.77 (0.43-1.43) |
| A (A) | 52 (55.32) | 37 (48.68) |  |  |
| **rs731236 (*Taq*I)** |  |  |  |  |
| Genotype |  |  |  |  |
| TT (TT) | 15 (31.91) | 15 (39.47) | 0.76 (0.48*) |  |
| TC (Tt) | 23 (48.94) | 17 (44.74) |  |  |
| CC (tt) | 9 (19.15) | 6 (15.79) |  |  |
| Allele |  |  |  |  |
| T (T) | 53 (56.38) | 47 (61.84) | 0.47 | 0.80 (0.44-1.51) |
| C (t) | 41 (43.62) | 29 (38.16) |  |  |
| **rs11568820 (Cdx2)** |  |  |  |  |
| Genotype |  |  |  |  |
| GG | 30 (73.17) | 25 (71.43) | 0.98 (0.85*) |  |
| GA | 10 (24.39) | 9 (25.71) |  |  |
| AA | 1 (2.44) | 1 (2.86) |  |  |
| Allele |  |  |  |  |
| G | 70 (85.37) | 59 (84.29) | 0.85 | 1.09 (0.46-2.52) |
| A | 12 (14.63) | 11 (15.71) |  |  |

* χ^2^ for trend test

AIT – autoimmune thyroiditis; OR – odds ratio; CI – confidence interval

**Table 2S.** Characteristic of AIT subgroups according to genotypes of rs1544410 (*Bsm*I) *VDR* polymorphism.

| **Genotype** | **GG** | **GA** | **AA** | **p** |
| --- | --- | --- | --- | --- |
| **Age** [years] | 48.47±13.15 | 47.00±14.87 | 42.85±12.77 | 0.34 |
| **Thyroid volume** [ml] | 6.79  (3.77-12.96) | 9.47  (5.52-19.60) | 10.96  (7.82-16.50) | 0.03 |
| **Follow-up time** [years] | 3.0 (1,5-7) | 2.0 (0-6) | 2.5 (1-4) | 0.35 |
| **Gender** (female/male) | 37/1 | 37/2 | 18/2 | 0.43 |
| **Age at diagnosis** [years] | 45.23±13.02 | 44.28±15.54 | 37.50±15.03 | 0.25 |
| **Levotyroxine dose** [µg] | 75.00  (75.00-100.00) | 87.50  (50.00-100.00) | 50.00  (50.00-100.00) | 0.18 |

Data are mean (standard deviation) or median (interquartile range)

**Table 3S.** Characteristic of AIT subgroups according to genotypes of rs7975232 (*Apa*I) *VDR* polymorphism.

| **Genotype** | **CC** | **CA** | **AA** | **p** |
| --- | --- | --- | --- | --- |
| **Age** [years] | 50.85±14.15 | 46.64±13.91 | 45.58±14.07 | 0.42 |
| **Thyroid volume** [ml] | 6.70  (3.34-13.42) | 7.61  (4.23-15.59) | 14.16  (9.02-17.84) | 0.04 |
| **Follow-up time** [years] | 3.0 (2-6) | 2.0 (0-7) | 3.0 (1-3) | 0.84 |
| **Gender** (female/male) | 19/1 | 46/1 | 21/3 | 0.17 |
| **Age at diagnosis** [years] | 50.77±11.37 | 42.03±14.88 | 41.05±15.97 | 0.14 |
| **Levotyroxine dose** [µg] | 81.50  (75.00-100.00) | 75.00  (50.00-112.50) | 75.00  (50.00-100.00) | 0.63 |

Data are mean (standard deviation) or median (interquartile range)
